# A fully automated liquid–liquid extraction system utilizing interface detection

**DOI:** 10.1155/S146392460000033X

**Published:** 2000

**Authors:** Eugene Maslana, Robert Schmitt, Jeffrey Pan

**Affiliations:** Abbott Laboratories Abbott Park IL USA

## Abstract

The development of the Abbott Liquid-Liquid Extraction Station was a result of the need for an automated system to perform aqueous extraction on large sets of newly synthesized organic compounds used for drug discovery. The system utilizes a cylindrical laboratory robot to shuttle sample vials between two loading racks, two identical extraction stations, and a centrifuge. Extraction is performed by detecting the phase interface (by difference in refractive index) of the moving column of fluid drawn from the bottom of each vial containing a biphasic mixture. The integration of interface detection with fluid extraction maximizes sample throughput. Abbott-developed electronics process the detector signals. Sample mixing is performed by high-speed solvent injection. Centrifuging of the samples reduces interface emulsions. Operating software permits the user to program wash protocols with any one of six solvents per wash cycle with as many cycle repeats as necessary. Station capacity is eighty, 15 ml vials. This system has proven successful with a broad spectrum of both ethyl acetate and methylene chloride based chemistries. The development and characterization of this automated extraction system will be presented.


Journal of Automated Methods & Management in Chemistry, Vol. 22, No. 6 (November–December 2000) pp. 187–194

### A fully automated liquid–liquid extraction system utilizing interface detection

**Eugene Maslana, Robert Schmitt and Jeffrey Pan**
*Abbott Laboratories, Abbott Park, IL, USA*

*The development of the Abbott Liquid–Liquid Extraction Station was a result of the need for an automated system to perform aqueous extraction on large sets of newly synthesized organic compounds used for drug discovery. The system utilizes a cylindrical laboratory robot to shuttle sample vials between two loading racks, two identical extraction stations, and a centrifuge. Extraction is performed by detecting the phase interface (by di erence in refractive index) of the moving column of fuid drawn from the bottom of each vial containing a biphasic mixture. The integration of interface detection with fuid extraction maximizes sample throughput. Abbott-developed electronics process the detector signals. Sample mixing is performed by high-speed solvent injection. Centrifuging of the samples reduces interface emulsions. Operating software permits the user to program wash protocols with any one of six solvents per wash cycle with as many cycle repeats as necessary. Station capacity is eighty, 15 ml vials. This system has proven successful with a broad spectrum of both ethyl acetate and methylene chloride based chemistries. The development and characterization of this automated extraction system will be presented.*

### Introduction

With the increasing implementation of automated organic synthesis systems, a greater burden had been placed upon the chemist to purify the resultant large numbers of synthesized compounds produced. As the quantities grew so did the amount of time the chemist had to spend at the bench performing rudimentary processes such as liquid separations. On 40 samples this wash process could take five or more hours to complete. As the sizes of compound libraries needed for drug development continued to grow, an automated system to free the chemist from this task was necessary.

This process readily lent itself to automation. However, to make such a system useful over a broad range of chemistries, an accurate and dependable method for detecting the boundary meniscus between the two solution phases was required. While volumetric based protocols have been common, they suffer from poor wash efficiency because of mass transfer between the organic and aqueous phases. Interface detection also had a clear advantage over other approaches since waste layer volume need not be known.

The choice of sensor technology was critical in the successful implementation of a sensor based method of detecting the interface. During the development of the Abbott system several different approaches were examined. One important consideration in the choice of detection schemes was the possibility of a large-radius meniscus at the interface which would not accurately indicate the interface between the layers. Optical scanning of the outside of the vial would not easily compensate for such a condition. Also it was only useful for chemistries with clearly defined interfaces having contrast between the layers. This approach was also rejected as being too slow for a high-throughput system since each vial had to be scanned individually and thus must be removed from a rack, increasing vial handling time. Electrical conductivity sensing, similar to fluid level detection methods, in commercial autosamplers was rejected as unreliable, particularly if the organic layer was on top. Other optical methods, such as a refractive index 'probe' that would be plunged into the solution until a change was detected, indicating the interface, were also rejected as unreliable.

Since our two primary goals during system development were accurate interface detection and high throughput, this necessitated both simultaneous detection and extraction as well as detection of the passing interface in the fluid stream during fluid extraction. By aspirating the fluid in a column significantly smaller in diameter than the vial, detection accuracy is increased as the passing meniscus is much smaller in radius. Therefore we chose to place a detector in line between the fluid probe (sample needle) and pump. This still required a sensitive and accurate method of detecting composition changes in the passing fluid. Refractive index was selected over conductivity sensing. Since we could not predict the nature of the passing interface we chose to measure the refractive index of the passing fluid stream at two different places and used the difference in the values as an indicator of the interface. This method helped to negate the effects of emulsions or dissolution of the interface during aspiration of the solution. We implemented this technique with a commercial differential refractometer in the fluid line between the fluid probe and fluid pump. By utilizing the refractometer output signal, with associated electronic signal processing circuitry and software, we were able to demonstrate accurate and reliable separations of biphasic solutions as they were drawn from their vials.

Depending on the chemistry involved and the technique employed, the result of liquid–liquid extraction can yield either a high recovery of the target compound with some contaminants or a high purity but with lower recovery. Often yield is low if the organic compound is water soluble. Whereas with many highly non-polar compounds, the chemist considers purity a greater issue than the total quantity produced and the numerous wash steps required to achieve this purity in turn lower the final yield. In the design of the station we had to provide the ability to perform extractions with either a high compound yield, requiring that extractions are performed on each successive waste layer, or a high purity with numerous washes on the organic layer. The high-yield mode was more difficult to implement as it required both saving of the waste layer for each wash step and swapping sample vials in order to perform the next wash on the preceding waste layer.

Once the detection method was realized we developed the automated system to perform all other tasks required in the extraction procedure. To maximize throughput we chose to have two identical extraction stations served by a single cylindrical laboratory robot and centrifuge, which reduced the overall cost of the station. The robot shuttles vials between the various components of the station.

### System description

As initially conceptualized, the basic requirements for the Liquid–Liquid Extraction Station were:

- accurate, must account for mass transfer between layers, sensor-based technology;
- high throughput, minimum of 100 samples processed per day;
- able to remove either the top or bottom layer of a biphasic solution;
- fully automated; and
- user friendly.

The Liquid–Liquid Extraction Station (figure 1) is designed to accept two racks of up to forty 15 ml vials each, add any one of six user selected solvents to the vials, transfer the vials to a centrifuge, spin the vials, return them to the pipetter, extract the waste layer and repeat this process for as many times as needed. The system is built around a Zymate XP Robot (Zymark Corporation, Hopkinton, MA) that shuttles the vials between the various devices on the deck of the system (figure 2).

### *Fluid system and pipettor*

The pipetters (AIM1250, A. I. Scientific, Scarborough, Qld, Australia ) are single tip, single arm autosamplers that are computer controlled via an RS-232 link. A removable vial rack mounts on the deck of each pipetter, which also contain wash wells for cleaning the probe between each extraction. The syringe pumps (XL3000, Cavro Scientific Instruments, Sunnyvale, CA) draw fluid from the bottom of each vial through the centre cannula of a custom dual-cannula probe designed by Abbott. Each probe contains an outer cannula for evacuation of the vial contents into a waste trap as well as dispensing fresh solvents into the vial, and an inner cannula is for drawing fluid into the refractometer. The probe's design ensures complete aspiration of the bottom layer with minimal mixing at the interface. Solvent selection is made through the operating system and is performed via an 8-port valve integral with each syringe pump. Fluid flow is controlled by a set of valves (figure 3). Pump and valves are computer controlled, the pumps through an RS-232 serial interface, the solenoid valves indirectly through a set of relay contacts provided by each pipetter.

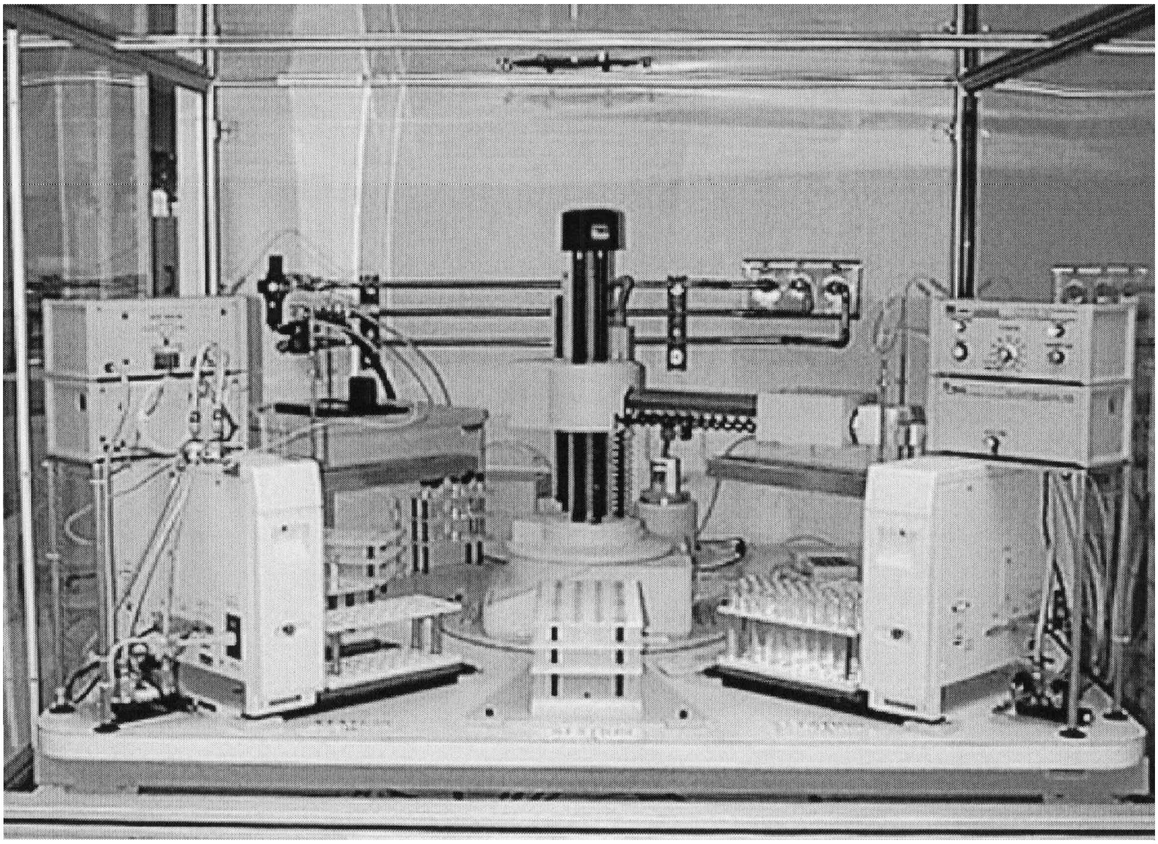

*Figure 1. The Abbott Liquid–Liquid Extraction Station.*

*Detector and interface electronics*

The differential refractometers (Model R403, Waters/ Millipore, Milford, MA ) contain two physically independent cells. Light passing through each cell falls on one of two photocells, comprising two halves of a bridge circuit. If the light intensity is not equal for the two detectors a signal is produced, varying proportionally with the difference in refractive index between the fluids in the two cells. The fluid path through the two cells is configured to be in series. Thus the first cell will always see a new fluid before the second. When a biphasic solution is drawn through the cells and the interface meniscus passes the first cell, it results in a large signal output from the detector's signal processing electronics (figure 4).

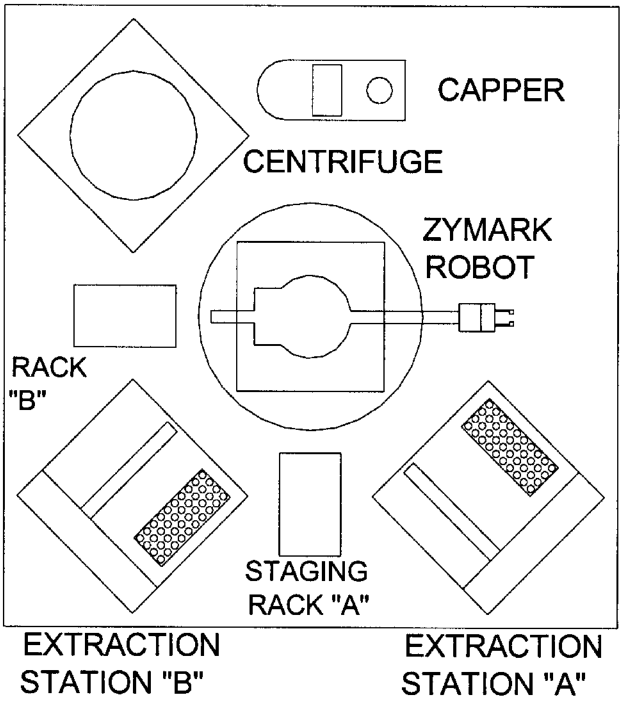

*Figure 2. System layout.*

The output signal of the refractometer is processed by circuitry designed and built by our group. The output signal is split, one signal line is amplified, the other low-pass filtered. The amplified, but not filtered, signal is passed through two offset amps, one high and one low. The filtered and the offset signals are then compared. If either the negatively offset signal rises more rapidly than the low passed filtered signal or the positively offset signal falls faster than the filtered signal a comparator goes high. A logic circuit 'OR's' the comparator outputs and this signal is ultimately sent to the output trigger circuit.

First, output from the 'differentiating' analogue section is sent to a digital 'window gate' controlled by the computer through a digital I/O board (Lab PC+, National Instruments, Austin, TX). The gate ignores the analogue signal at all times except during detection operations. It is enabled by the digital I/O board only after the computer has determined that the syringe pump has moved a predetermined number of steps (this prevents detection of the bottom layer entering the detector). When the passing interface is detected by the 'differentiating' signal processing circuit the resultant TTL trigger pulse sent to the computer through the digital I/O board stops the syringe pump from drawing any more fluid through the detector.

*Shuttle robot and centrifuge*

The robot is used to shuttle the vials to and from the staging racks, the pipetters and the centrifuge (Model ZP710-2, Zymark). The centrifuge works in concert with the robot to maintain the order of the vials as they are placed in it. A special drive system indexes the centrifuge rotor to a specific position for the robot to place or remove a particular vial. The capacity of the centrifuge is 24 vials. If there are greater than 24 samples to be processed the centrifuge step is performed in two batches. A rack of 'balance' vials, with varying weights, is provided. If an odd number of vials is to be spun the

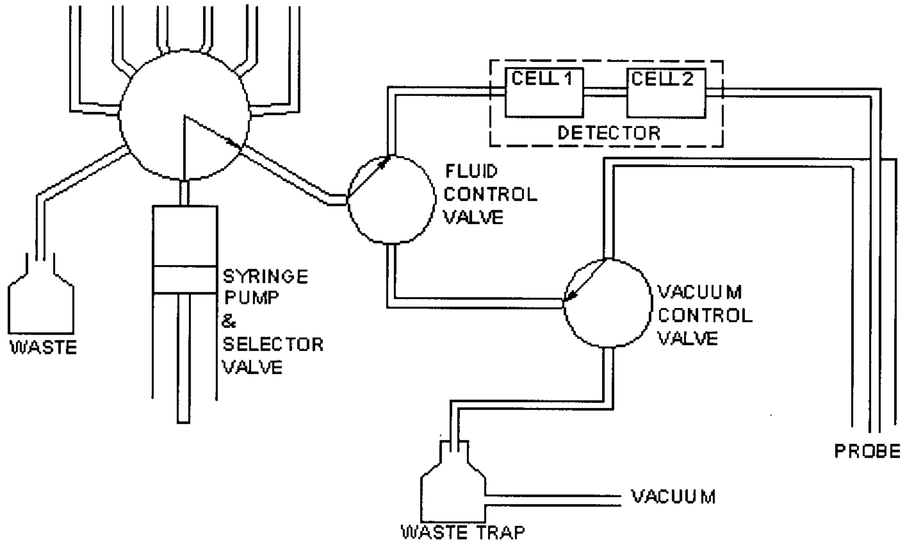

*Figure 3. Fluid circuit for one station.*

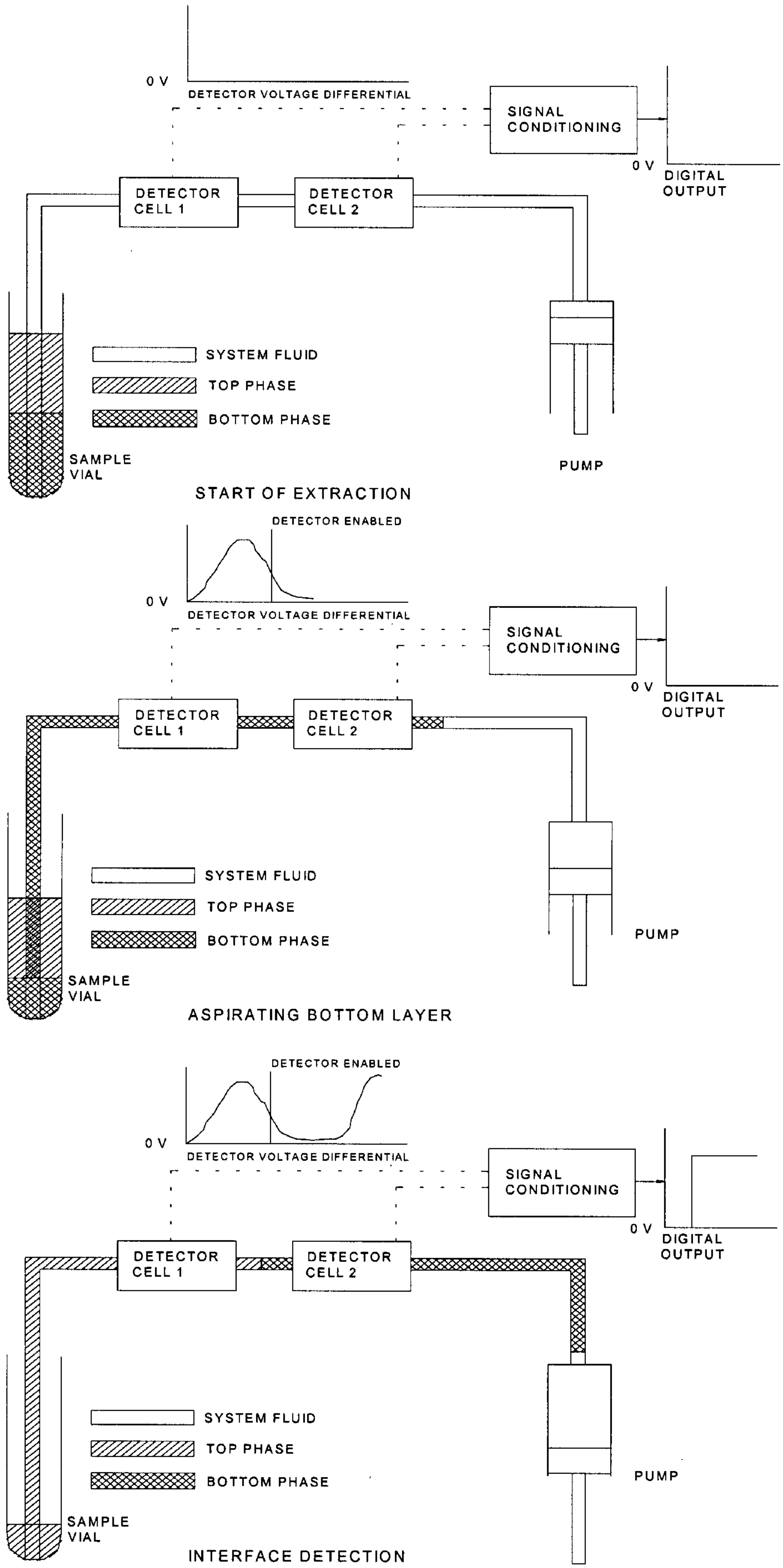

*Figure 4. Interface detection process.*

appropriate balance vial is selected automatically and placed in the centrifuge to balance the load. Since the balance vials are weighted with water, and thus capped, a vial capper (Model ZP410, Zymark) removes the cap before the vial is placed in the centrifuge and replaces it when done. The robot, centrifuge and capper are controlled by a control unit (System V Controller, Zymark) that is an interface between the operating system PC and the three devices. The controller is linked to the system control PC by an RS-232 serial interface.

*System operating software*

The software operating system is comprised of four parts: operator interface (for wash protocol set-up), device command code parser with logic control, Zymark System V control programming, and device command (script) files. The operator interface and command code parser are written in Microsoft Visual Basic (VB) v. 4. The operator interface allows for easy set-up of extraction protocol parameters such as type of extraction, the number of samples, solvent volume, and number of washes. An extraction protocol is created through a window that defines each step of the extraction process, i.e. wash solvent, which layer is the waste, etc. (figure 5).

The resultant file is generic and can be used for any number of vials and solvent volumes (within-operating limits of the station). Less-used system operating parameters, such as pump speed and minimum volume aspirated before detection, may also be adjusted by the user through an appropriate window (figure 6).

A particular wash protocol is selected and executed through a 'Run Set-Up' screen. This screen gives the user the ability to set volumes and number of vials (figure 7).

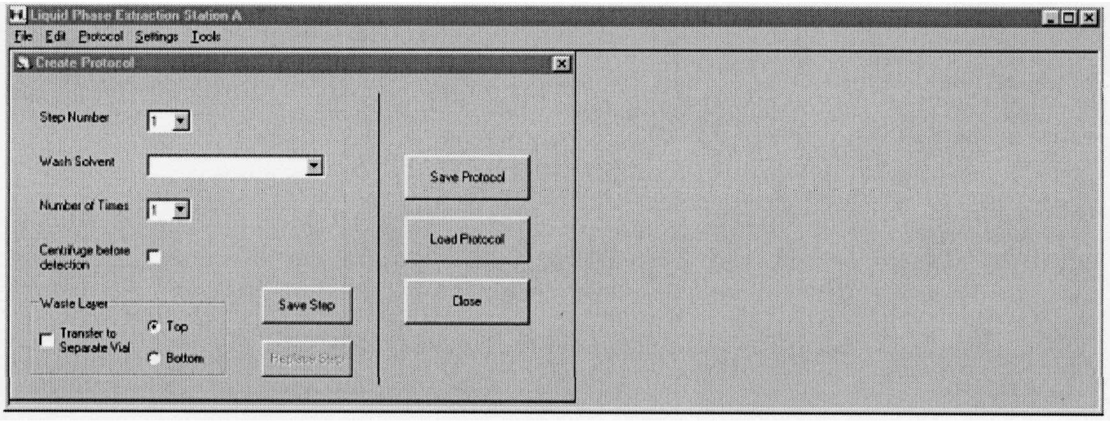

*Figure 5. Protocol set-up screen.*

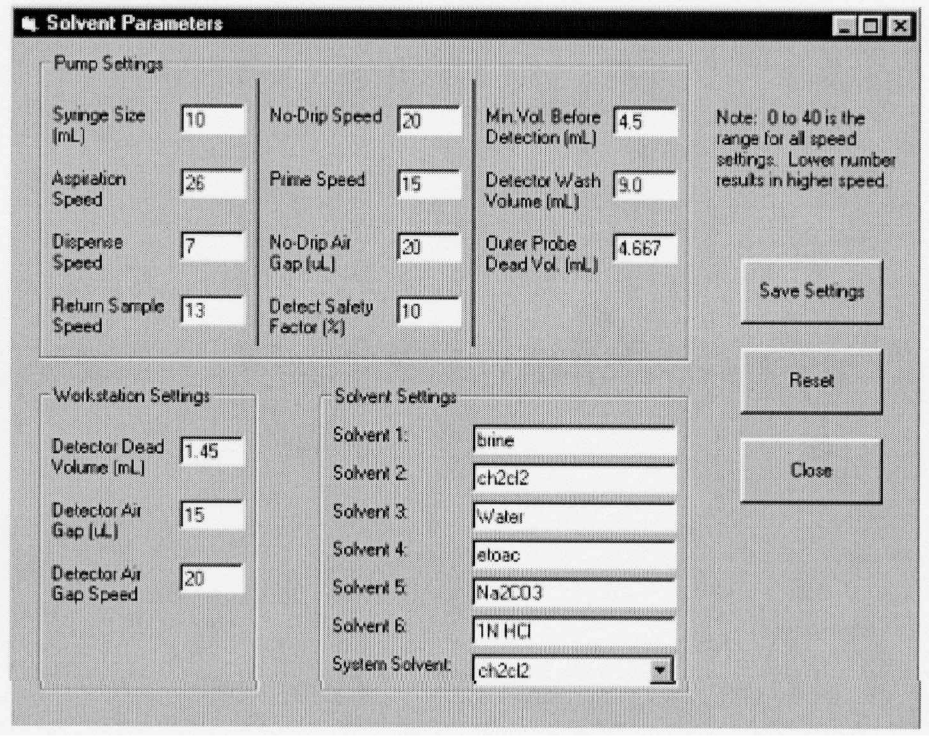

*Figure 6. System parameters.*

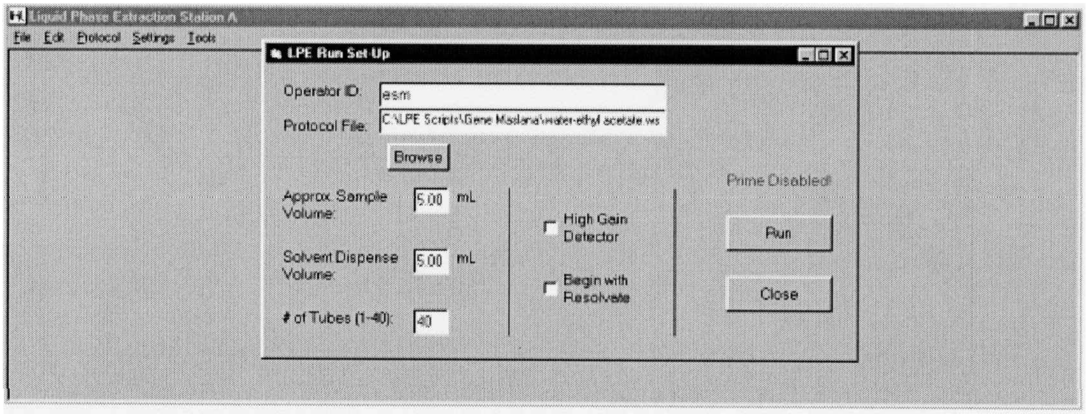

*Figure 7. Run Set-Up screen.*

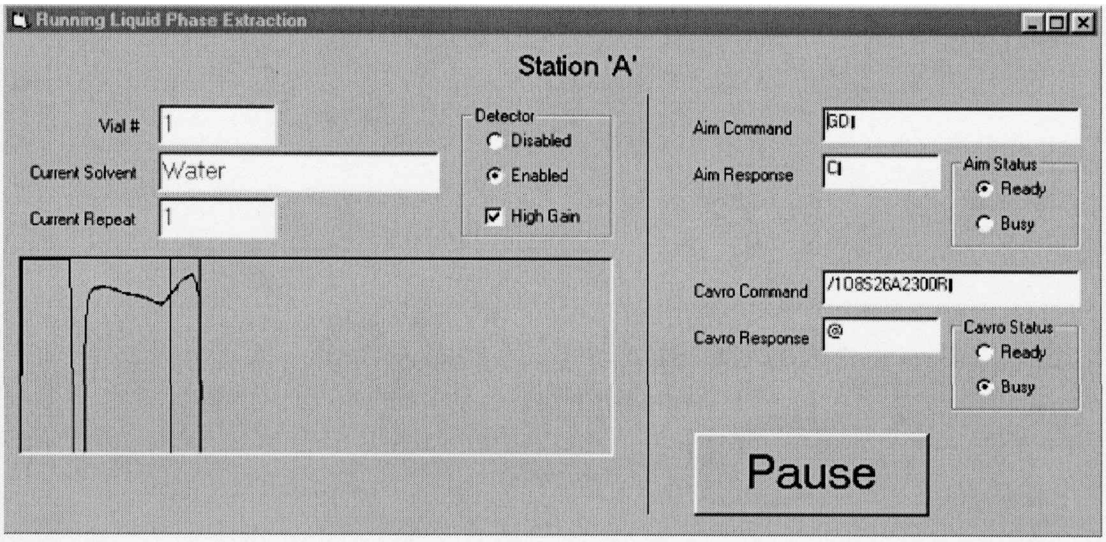

*Figure 8. Station status screen.*

During operation of the station a status screen is displayed which indicates the current vial, wash solvent, and device commands. Extraction status is also indicated by a graph of the differential refractometer output and an indicator line showing the point in time of interface detection (figure 8).

The user interface modifies pre-written command scripts (device command files in ASCII text) which are the sequential commands that perform particular tasks such as solvent addition or extraction. The user interface rewrites commands as needed, depending on the values of selectable parameters. Underlying this interface is a system control program or 'script engine' that assembles the various scripts into one large file that is all the commands needed to perform the extraction protocol. The script engine then parses the command code to the non-Zymark devices as well as variables to the EasyLab programs stored in the System V controller (figure 9). Therefore the same set of command scripts can be used for any number of extraction protocols without the need to write protocol-specific extraction programs. The command code parser also performs the necessary logic and control needed to operate the pump during extraction. It activates the detector interface unit and monitors the unit's output for an 'interface detected' signal. To operate the Zymark robot, centrifuge and capper, custom software was supplied by Zymark for their EasyServ OLE Server that interfaces the Abbott Visual Basic operating system with the Zymark System V Controller. This software allows for easy parsing of operating variables between the System V Controller and the Visual Basic system and permits the Zymark devices to operate in the 'background' while the two extraction stations can continue with their tasks.

This approach to instrument control software has two significant advantages. First, it separates the device-specific control commands (i.e. what is sent via the RS-232 links to each device) from the user interface, enabling easy modification of the operating sequence of the station without having to modify the user interface code that is written in Visual Basic. Since the command scripts are simply device commands that are strung together in the sequence of operation for a particular task, the user can change an operating sequence by just editing the script file in an ASCII text editor such as Notepad. It also makes for a more 'portable' software package that can be utilized in other applications. Since the Visual Basic operating system will parse any ASCII text code, this

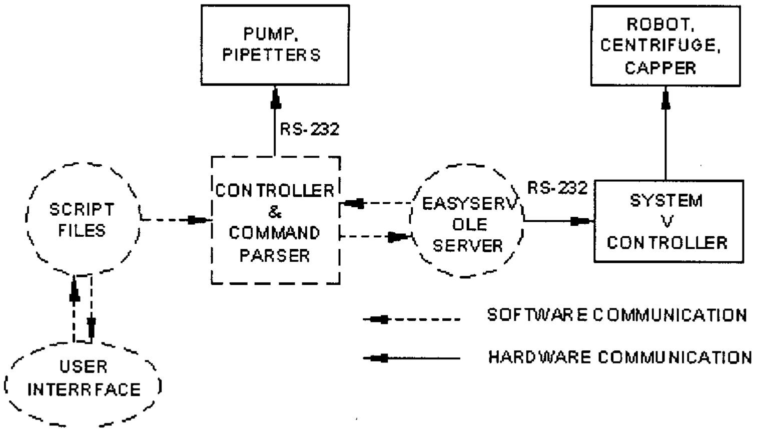

*Figure 9. Control schematic.*

software can be used for other applications (in fact it has been the basis for many such types of control program).

*System operating procedure*

The basic operation of the station is as follows. The operator chooses which station, 'A' or 'B', to use and loads the sample vials into the corresponding staging racks. Both stations can be used simultaneously with different wash protocols. The appropriate wash solvents are loaded. The wash protocol(s) are written or loaded, any other settings necessary for the procedure are entered. The procedures for the station(s) are initiated. The robot transfers the vials to the appropriate extraction station. Solvent is added to each vial by high-speed injection to adequately mix the solutions. The vials are transferred to the centrifuge and spun for the selected time. The vials are returned to the extraction station. Extraction is performed on each vial. This procedure is repeated for as many times as required. Once the last extraction step is complete, the sample vials are returned to the staging racks.

### System validation and performance

During development of the station we conducted several experiments to test the efficiency and accuracy of the extraction system. Our concern was both the efficiency of waste removal from the compound layer (with minimal compound loss) as well as contamination from carryover between vials.

*Mass recovery and compound carryover test*

Ten vials each with 25 mg Indomethacin Weinreb amide dissolved in 5 ml ethyl acetate and ten with 50 mg boc-6,7-dimethoxytetrahydroisoquinoline also in 5 ml of ethyl acetate were alternated in the vial rack. Three washes were performed, comprising a 5 ml addition of water and centrifuging for 5 minutes. After the final wash the solvents were evaporated in a SpeedVac (Savant Instruments, Farmingdale, NY) and the compounds were weighed. Thin layer chromatography (TLC) was performed to check for unwanted compounds.

*Table 1. Compound recovery, prototype system.*

| | Recovery ± SD |
|---|---|
| Boc-6,7-dimethoxytetrahydroisoquinoline | 67% ± 15% |
| Indomethacin Weinreb amide | 86.8 ± 7.6% |

For the 25 mg set of vials containing the Indomethacin Weinreb amide the average final weight was 21.7 mg with a standard deviation of ±1.89 mg. This resulted in a recovery of 86.8% ± 7.6% (table 1). The boc-6,7-dimethoxytetrahydroisoquinoline vials contained an average of 33.4 mg with a standard deviation (SD) of ±7.5 mg. This was a recovery of 67% ± 15%. Two-solvent TLC of the 20 vials showed only single traces for each vial, indicating a purity of greater than 95%.

*Compound wash test*

A second test of the ability of the system to separate actual compounds was performed with compounds synthesized at Abbott. Two different sets of unpurified organic compounds (6 compounds and 10 compounds) solvated in methylene chloride were extracted. The same wash protocol of two citric acid washes, two sodium carbonate washes, and two water washes with 5 minutes of centrifuging for each step was used on both sets. Purity ranged from 61% to 99% (average 89.6% ± 9.0%). This procedure was typical of most wash protocols performed on the station and was in high-purity mode, i.e. many washes of only the organic layer without any extraction performed on the waste layer.

*System performance with typical extraction protocols (high-purity mode)*

A typical extraction protocol was performed on 20 vials each containing 60 mg z-beta-alanine dissolved in 5 ml methylene chloride. The wash protocol consisted of four steps (two of 1N HCl, one of water, one of brine) each of 5 ml volume. One additional sample (not washed) was kept as a control. Samples were then dried in a SpeedVac and weighed. A second test with five vials containing 60 mg chloromethylanthracene also dissolved in methy-

*Table 2. Compound recovery, automated system.*

| Compound | Recovery ± SD |
|---|---|
| z-beta-alanine | 41% ± 3% |
| Chloromethylanthracene | 53.8% ± 6.6% |

lene chloride was conducted. The same protocol was performed (table 2).

An additional test was performed to test cross-contamination of samples. Again 20 vials of 60 mg z-beta-alanine in 5 ml methylene chloride were alternated in the rack with 20 vials of boc-alanine, 60 mg in 5 ml methylene chloride. The same wash protocol of HCl, water and brine was performed. HPLC analysis of the final washed compounds showed no detectable trace of the adjacent compound in any of the vials.

The procedure times for a typical extraction are presented and compared with the time to perform the same extraction by hand. If one extraction station performs a four-wash extraction the time is only one hour slower than if it were done by hand. However, if both stations are used, with 20 samples on each station, the time is cut nearly in half and is only 67% of manual extraction time (table 3).

### Conclusion and summary

The test results show that the Liquid–Liquid Extraction Station is effective at extracting waste products from solubilized compounds with good product yield and no contamination. Compound purity of 99%, of previously synthesized organic compounds, is achievable with no detectable cross-contamination from compounds in adjacent vials. Compound recovery is acceptable, and can be enhanced in the case of highly polar compounds by operation in a high-yield mode. Throughput for the station compares favourably with manual extraction processing performed by chemists at the bench. The ability of the station to purify compounds has proven satisfactory to all users and compound yield has only been a concern in some rare instances with certain types of chemistries when the 'high-yield' mode was used. The station has been able to handle a broad spectrum of chemistries with precipitates and emulsions rarely having an effect on the station's ability to properly detect an interface and perform an extraction.

The fully automated liquid–liquid extraction station has both increased the efficiency of our combinatorial chemists and reduced their workload. In 18 months of operation the station has proven to be reliable in operation, processing over 6000 compounds.

### Acknowledgements

The authors gratefully acknowledge the following individuals without whose help this station would not be a reality: Abbott Laboratories—Guy Clark, Jack Conrad, Lisa Frey, Stefan Loren, Linda Lynch, Michael Routburg; and Zymark Corporation—Tom Kinzelman.

### Trademarks

Zymark, Zymate, Easylab, and EasyServ are either trademarks or registered trademarks of Zymark Corporation. Visual Basic is a registered trademark of Microsoft Corporation. SpeedVac is a registered trademark of Savant Corporation.

*Table 3. Comparison of process times for sample extraction.*

| | Extraction time, four wash steps | | |
|---|---|---|---|
| Number of vials in procedure | One station only | Stations A and B | Manual extraction |
| 40 vials | 7 hours | 4 hours | 6 hours |
| 80 vials | Not applicable | 8.5 hours | 8 hours |

